# Response to Hydroxyurea in a Patient With Sickle Cell Hepatopathy: A Case Report

**DOI:** 10.7759/cureus.20649

**Published:** 2021-12-23

**Authors:** Maha A Safhi, Rana M Baghdadi, Adel F Al-Marzouki, Fatin Al-Sayes

**Affiliations:** 1 Medicine, King Abdulaziz University Faculty of Medicine, Jeddah, SAU; 2 Internal Medicine, King Abdulaziz University Faculty of Medicine, Jeddah, SAU; 3 Hematology, King Abdulaziz University Faculty of Medicine, Jeddah, SAU

**Keywords:** exchange blood transfusions, sickle cell intrahepatic cholestasis, sickle hepatopathy, sickle cell disease, hydroxyurea

## Abstract

Sickle cell hepatopathy is an underreported entity lacking clear management guidelines. This case highlights the potential role of hydroxyurea (HU) in improving the hepatic dysfunction seen among patients with sickle cell disease (SCD). We herein present the clinical course of a patient prior to and after the initiation of hydroxyurea with an emphasis on long-term outcomes and the patterns of liver injury over a 15-year time course.

## Introduction

Sickle cell disease (SCD) is a hemoglobinopathy with multisystem involvement and a variety of hepatobiliary manifestations [1]. Sickle cell hepatopathy encompasses the various patterns of liver injury seen among patients with SCD. The causes of disturbed liver functions among patients with SCD vary from the sequelae of intrahepatic sickling and cholestasis due to the primary disease process to the various comorbidities associated with the disease course and its treatment [2,3]. The disease spectrum involves ischemic injury secondary to the sickling process, pigmented gallstones, and acute and chronic hepatic sequestration [4], leading to a spectrum of manifestations that range from benign hyperbilirubinemia to overt liver failure [1]. Sickle cell intrahepatic cholestasis (SCIC) is usually described as an acute or recurrent entity but may be a harbinger for chronic liver disease [3]. A well-established treatment regimen is based on exchange blood transfusions (EBTs). Hydroxyurea (HU), the antineoplastic agent known to combat life-threatening complications such as acute chest syndrome and stroke, has been used very sparingly in sickle hepatopathy [4].

## Case presentation

The patient is a 30-year-old female known to have had sickle cell disease (SCD; HbSS) since early childhood. She began to follow up at our institution in 2007 at the age of 16. She had not been on regular transfusion programs or hydroxyurea prior to this point. Initially, she was on folate supplements and analgesics as needed. She refilled monthly prescriptions of morphine 30 mg three times per day and tramadol 50 mg three times per day every two to three months for use during painful crises. She had a severe disease course characterized by frequent emergency room visits and hospital admissions secondary to pain crises. Her history was notable for frequent but irregular simple blood transfusions and a cholecystectomy at 12 years old.

**Figure 1:**
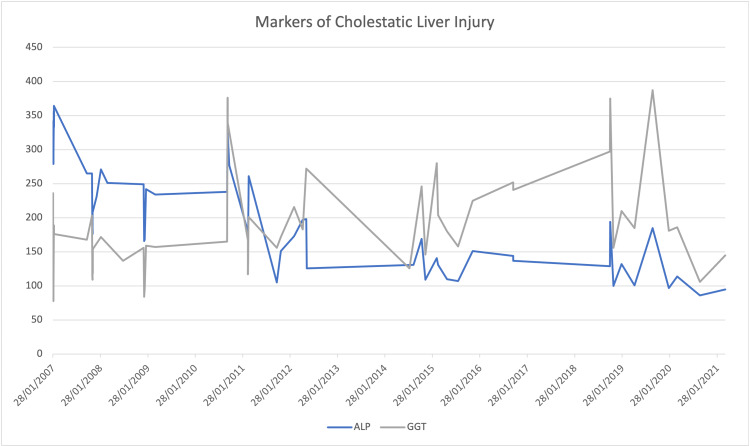
Progression of alkaline phosphatase and gamma-glutamyl transferase prior to and following hydroxyurea initiation in 2015

**Figure 2:**
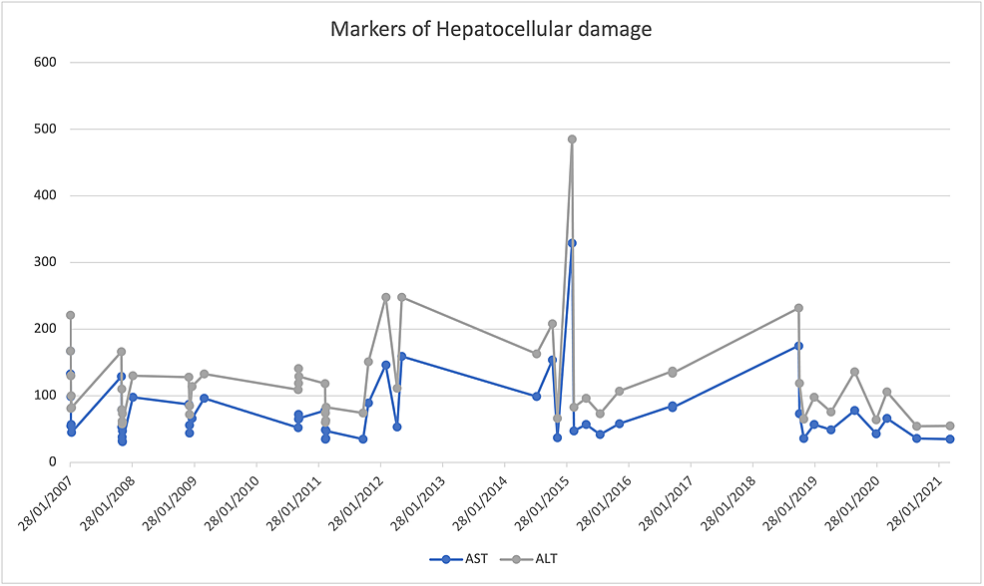
Pattern of aspartate aminotransferase (AST) and alanine aminotransferase (ALT) from 2007 to 2021

Clinically, her complaints often centered around vaso-occlusive musculoskeletal pain. However, on presentation, she displayed persistently disturbed liver function tests (LFTs) (Figure 1 and Figure 2): alkaline phosphatase (ALP), 301 U/L; gamma-glutamyl transferase (GGT), 236 U/L; aspartate aminotransferase (AST), 167 U/L; alanine aminotransferase (ALT), 54 U/L; total bilirubin, 426 umol/L; and direct bilirubin, 237 umol/L.

**Figure 3:**
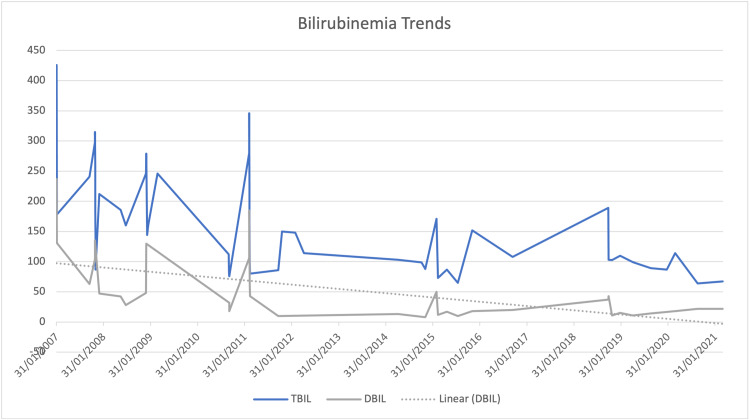
Total and direct bilirubinemia trends from 2007 to 2021

Figure 3 portrays persistent hyperbilirubinemia with an elevated direct component, accompanied by elevated markers of cholestasis (Figure 1). Elevations in liver transaminases were less pronounced but peaked during an acute episode in 2015. Synthetic functions remained unaffected, as both INR (1.2) and albumin (42 g/L) were within normal ranges. During her hospitalization, the patient underwent thorough investigations to investigate the possible causes of hepatic dysfunction.

**Figure 4:**
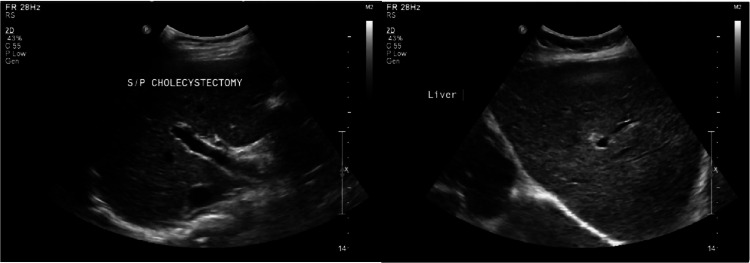
Ultrasound of the liver showing homogeneous echotexture and unremarkable postcholecystectomy biliary anatomy

Viral serology panels for viral hepatitis and human immunodeficiency virus (HIV) were negative. Imaging, including an ultrasound of the liver, confirmed the patency of her biliary tree (Figure 4). The liver was enlarged at 17.5 cm in the longitudinal dimension but otherwise had a homogeneous echotexture with no focal lesions. Comparisons to older images showed preexisting mild hepatomegaly without significant changes in the size or echogenicity of the liver. The common bile duct and intrahepatic biliary tree were noted to be of normal caliber and course.

Serum ferritin peaked at 900 ug/L. Furthermore, antinuclear antibody (ANA) was weakly positive, with a titer of 1:80. However, antimitochondrial antibody and smooth muscle antibody were both negative, ruling out autoimmune hepatitis. Ultimately, the elevated total bilirubin levels were not explained by severe acute hemolysis, viral hepatitis, extrahepatic obstruction, obstruction, or hepatic sequestration. With a BMI of 24.61 and a balanced diet that excludes any alcohol intake, nonalcoholic steatohepatitis (NASH) and alcoholic liver disease (ALD) were also ruled out. In the absence of suggestive serologies, a biopsy of the liver was not pursued.

She was labeled as a case of “sickle hepatopathy” as per Ahn et al.’s definition [1]. Based on her persistent symptoms and hepatic dysfunction, she was initiated on a regular exchange transfusion program every six weeks for one year. After failing to respond both clinically and biomedically, hydroxyurea was initiated at a dose of 1000 mg daily. The trend after 2015 shows a progressive decline in direct bilirubinemia. Her emergency room visits and hospital admissions secondary to hepatic and vaso-occlusive crises significantly dropped from four to six per year to one to two per year, and she reports an improvement in pain-related events. The patient continues to follow up with regular outpatient visits, the last of which took place in October 2021. She reports no new complaints or symptoms suggestive of liver damage. Her most recent laboratory results were as follows: hemoglobin, 7.9 g/dL (baseline: 7-9 g/dL); mean cell volume (MCV), 114.4 fL; white blood cell (WBC) count, 8.04 K/uL; platelet (PLT) count, 226 K/uL; albumin, 43.5 g/L; ALP, 122 U/L; AST, 52 U/L; ALT, 29 U/L; GGT, 153 U/L; total bilirubin, 92 umol/L; direct bilirubin, 15 umol/L; and ferritin, 118 ng/mL. The macrocytosis relative to her baseline MCV is reflective of good compliance.

## Discussion

Labeling a patient as a case of sickle cell hepatopathy is achieved by establishing hyperbilirubinemia > 200 umol/L (12 mg/dL) in the absence of hyper-hemolysis and confounding etiologies that may be directly responsible for disturbed liver functions [1]. The biochemical pattern may vary and often reflects an amalgamation of underlying abnormalities, including intrinsic liver disease, biliary cholestasis, and active hemolysis. Synthetic functions are relatively preserved [5]. Therefore, diagnosing this entity necessitates serology to rule out infectious hepatitis and an autoimmune panel to detect coexisting autoimmune hepatitis, primary sclerosing cholangitis (PSC), or primary biliary cirrhosis (PBC). Further workup involves imaging to assess for vascular compromise, patency of the biliary tree, and fatty liver via liver ultrasound and magnetic resonance cholangiopancreatography (MRCP), endoscopic retrograde cholangiopancreatography (ERCP), and CT/MRI of the abdomen as indicated. Investigating for secondary hemochromatosis is particularly relevant among patients on regular transfusion programs. It is also imperative to rule out toxic ingestions, including alcohol and drug history, while specifically eliciting any use of hepatotoxic drugs, including analgesics. This workup may reveal biliary disease that is managed surgically or endoscopically, iron overload that requires chelation therapy, or other etiologies that can be managed with targeted therapies. Otherwise, patients are managed with supportive measures such as hydration, analgesia, and transfusions as required.

The role of EBT in the treatment of sickle cell hepatopathy has been well established with favorable results. Ahn et al. documented reduced mortality among patients receiving EBT among those with mild and severe hepatic dysfunction [1]. Similarly, Khan et al. reported a case of severe sickle hepatopathy compounded by renal dysfunction, which was resolved following EBT with a target of HbS < 20% [6]. Malik et al. reported a similar presentation that responded to the EBT of nine units. Other cases with dramatic presentations and persistent end-organ dysfunction secondary to fulminant liver failure have necessitated liver transplant with favorable outcomes posttransplantation [7].

Hydroxyurea, on the other hand, has not been well explored in the context of sickle cell hepatopathy. While it can be extrapolated that the exact mechanisms responsible for reduced vaso-occlusive pain, acute chest syndrome, and decreased transcranial doppler measurements, among other complications, would also decrease the incidence of sickle cell hepatopathy, no studies have been carried out to confirm this hypothesis. Hydroxyurea acts on a cellular level to alter the kinetics of hemoglobin production in favor of fetal hemoglobin [8]. This is postulated to reduce the propensity of red blood cells to undergo sickling in the low oxygen tension environment within hepatic sinusoids. Moreover, the resultant decrease in the hemolysis rate diminishes the bilirubin load that contributes to intrahepatic cholestasis. Serial liver biopsies were taken from a patient with chronic hepatic sequestration to document the improvement of sinusoidal sickling and congestion following the initiation of hydroxyurea therapy [9]. While no histopathological evidence of improvement was obtained in our patient, it may be construed that her improvement is attributed to similar mechanisms.

## Conclusions

Our patient’s response to hydroxyurea resulted in fewer vaso-occlusive episodes, improved pain management, and improved hepatic function although she had failed a trial of short-term regular EBT. While this offers good insight into the potential role of hydroxyurea in the treatment of sickle cell hepatopathy and the evasion of EBT-related complications, more cases need to be documented to corroborate the relationship between HU and LFT normalization.
